# Poverty concentration in an affluent city: Geographic variation and correlates of neighborhood poverty rates in Hong Kong

**DOI:** 10.1371/journal.pone.0190566

**Published:** 2018-02-23

**Authors:** Yingqi Guo, Shu-Sen Chang, Feng Sha, Paul S. F. Yip

**Affiliations:** 1 Department of Social Work and Social Administration, The University of Hong Kong, Pokfulam, Hong Kong SAR, China; 2 Institute of Health Behaviors and Community Sciences and Department of Public Health, College of Public Health, National Taiwan University, Taipei, Taiwan; 3 Hong Kong Jockey Club Centre for Suicide Research and Prevention, The University of Hong Kong, Hong Kong SAR, China; Rice University, UNITED STATES

## Abstract

Previous investigations of geographic concentration of urban poverty indicate the contribution of a variety of factors, such as economic restructuring and class-based segregation, racial segregation, demographic structure, and public policy. However, the models used by most past research do not consider the possibility that poverty concentration may take different forms in different locations across a city, and most studies have been conducted in Western settings. We investigated the spatial patterning of neighborhood poverty and its correlates in Hong Kong, which is amongst cities with the highest GDP in the region, using the city-wide ordinary least square (OLS) regression model and the local-specific geographically weighted regression (GWR) model. We found substantial geographic variations in small-area poverty rates and identified several poverty clusters in the territory. Factors found to contribute to urban poverty in Western cities, such as socioeconomic factors, ethnicity, and public housing, were also mostly associated with local poverty rates in Hong Kong. Our results also suggest some heterogeneity in the associations of poverty with specific correlates (e.g. access to hospitals) that would be masked in the city-wide OLS model. Policy aimed to alleviate poverty should consider both city-wide and local-specific factors.

## Introduction

The geographic concentration of urban poverty has become one of the most important social issues in many contemporary metropolitan areas [1]. The dramatic increase in the number of residents living in high-poverty neighborhoods between 1970 and 1990 has attracted much attention [2]. Over the past three decades, researchers have widely debated both the causes and consequences of concentrated urban poverty. The explanations of urban poverty highlighted by scholars typically include economic and social restructuring [3], racial segregation [4], demographic structure [5], and public policy [6].

Most previous studies have focused on identifying urban macro-level economic and social processes that may universally contribute to poverty concentration. Although such processes may take different forms in different urban areas [2], previous investigations have mostly used geographic methods based on the assumption that the association between neighborhood poverty and correlates are spatially stationary [3, 4, 7]. It is important for policy makers to identify the local-specific patterns of these associations and implement tailored strategies for different neighborhoods that address the urban poverty issue. Moreover, most previous urban poverty research has been conducted in Western settings, especially in metropolitan areas in the United States [3, 4, 8, 9]. Little is known about whether a similar pattern of urban poverty and locally specific characteristics could be found in the Asian context. The fact that Asia is experiencing rapid economic restructuring and urbanization highlights the need for more urban poverty research in Asian settings [10].

Hong Kong, a former British colony and a Special Administrative Region of China since 1997, is one of the most crowded places in the world, with 7.1 million people (2014) living in only 7% of a total area of 1,104 square kilometers (SI:1.104 billion square meters) [11]. Given the high level of economic inequality and the different social, cultural, and historical contexts that distinguish Hong Kong from Western metropolitan areas, we hypothesized that the characteristics of high-poverty neighborhoods in Hong Kong would differ from those seen in Western cities. The aims of this study were to: 1) investigate the spatial patterning of neighborhood poverty; 2) identify areas with concentrated poverty; 3) explore the city-wide and local-specific correlates of neighborhood poverty; and 4) reveal different forms of poverty concentration in Hong Kong. These results will be of interest and relevance to many high-income Asian cities that are experiencing rapid demographic, social, economic transformations.

## Research context

The literature examining factors associated with neighborhood poverty is extensive. Two dominant explanations for neighborhood poverty typically offered by scholars are economic restructuring and class-based segregation [2, 3, 9] and racial segregation [4, 12, 13]. Besides these two arguments, several recent works have highlighted the role of demographic structure [5, 14] and public policy [6, 15–20] on the spatial patterning of urban poverty.

### Economic restructuring and class-based segregation

An overarching theme in many previous studies of urban poverty is economic restructuring and accompanied class-based segregation. These studies mainly follow Wilson’s (1987) famous work *The Truly Disadvantaged*. Using data from Chicago, Wilson [3, 21] argued that the increasing concentration of urban poverty is a reflection of contemporary economic restructuring and the accompanying residential mobility of the middle class. Some studies have tested this theory and come to similar conclusions. For example, Kasarda [22, 23] has discussed how the combined forces of deindustrialization, occupational bifurcation, and the suburbanization of employment opportunities have isolated poor urban residents. Kasarda [22] argued that deindustrialization resulted in a decrease in manufacturing jobs for poorly educated residents in inner-city areas. Many jobs were moved out from inner-city areas to suburban areas. Compounding the economic restructuring is an increase in class-based residential segregation. Middle-class flight from poor inner-city neighborhoods to suburban areas has left poorly educated residents economically and socially isolated, causing an increase in poverty concentration along with a social phenomenon that labels the persons who live in high-poverty neighborhoods as “urban underclass”.

### Racial segregation

A second line of inquiry into urban poverty highlights the role of racial segregation. Scholars have argued that racial segregation also plays a significant role, no less than class-based residential segregation, in explaining concentrated poverty [4, 12, 24]. For example, the work which opened this discussion was *American Apartheid* by Massey and Denton [4], who argued that increasing poverty concentration is a reflection of both economic inequality and racial segregation, and that racial segregation reflects urban neighborhood poverty more than economic inequality. Zubrinsky [13] supported Massey and Denton’s argument and showed that economic differences themselves were insufficient to explain the variation in neighborhood poverty based on data from four cities in the United States. Squires [25] also noted that racial segregation contributed to uneven urban development in metropolitan areas. Finally, in *Poverty and Place*, Jargowsky [8] conducted a comprehensive analysis investigating urban census tracts data in the United States and concluded that the arguments put forward by Wilson [3] and Massey and Denton [4] were not mutually exclusive.

### Demographic structure and public policy

Several recent studies explored other factors shaping neighborhood poverty, such as demographic structure and public policy. For example, Jargowsky [8] found that one of the major features of areas with high poverty concentration is a higher proportion of female-headed households. Benson et al. [5] and Curtis et al. [14] highlighted the significant association between demographic situations (e.g. vulnerable groups who have limited earning potential, dependency ratio, and population density) and poverty rate. Carter, Schill [6] found a positive association between public housing and neighborhood poverty in four cities in the United States. Another group of scholars noted that the combined barriers of inaccessible public transport, long commuting time, and high transport fares prevent residents with less income, lower education, and unprofessional skills from attaining jobs [16–18]. Swanstrom, Dreier [20] argued that public health services in most cities are supplied by local government and high-poverty areas are more likely to lack fiscal capacity, therefore high-poverty areas often receive notably different services than other areas. A lack of services in turn exacerbates the situation in high-poverty areas.

### Spatial variation of association

Although past research has attempted to identify determinants or correlates of urban poverty using various approaches, one important similarity in most studies is that they emphasize the development of a generalized explanation across all locations but neglect to consider fully how poverty concentration may take different forms in different places. A generalized explanation may not be appropriate in specific metropolitan areas and, more importantly, may misguide public policies because it does not fully consider specific local processes [2, 5, 7, 14]. Although researchers have long been aware of the role of specific locations, few empirical studies have investigated different forms of concentrated poverty in metropolitan areas. The lack of locally specific analysis may be largely explained by a lack of appropriate analytical tools, while recent advances in geographically weighted regression (GWR) now allow for such analysis [26]. For example, Longley [27] investigated the spatial pattern of neighborhood hardship and its correlates at both city-wide and specific-local levels by applying GWR, and confirmed that urban neighborhood hardship takes different forms in different urban areas. [3, 4, 8, 10]

### Unique features of Hong Kong

Besides the major political change after returning to China, Hong Kong experienced two other significant transitions during the late 1990s: 1) shifting from a manufacturing-dominated economy to a service-led economy; and 2) population decentralization to the New Territories [28]. This economic shift led to impressive GDP growth and Hong Kong became one of the most affluent cities in the world, with GDP per capita of US$53,000 in 2014. However, Hong Kong has also witnessed a widening income gap between the rich and the poor in recent decades, which has also been reflected in the spatial residential pattern [29]. Compared with the Western context, especially the United States, racial difference is less a significant social phenomenon in Hong Kong. Instead, immigrants from mainland China are playing a crucial role in shifting Hong Kong’s population structure [30]. Hui, Li [30] investigated the residential patterns of mainland immigrants in Hong Kong and noted that although they share the same racial/ethnic background, new Chinese immigrants also present particular residential patterns that can be identified. Public housing is an important poverty alleviation policy in Hong Kong, which has a long history of building public housing dating back to 1953 [29]. Delang and Lung [29] explored the impact of public housing on poverty concentration and found that public housing does not necessarily concentrate poverty. However, their analysis was based on data from the 1990s and their geographic scale, the tertiary planning unit (TPU), is very large, comprising roughly 30,000 residents, six times bigger than the census tract in the United States. In terms of other public policies, the Hong Kong government is known for its dedication to building a fairer society. However, few empirical studies have been conducted to examine whether public services are evenly allocated across different areas.

## Methods

### Data

We used data from the 2011 Hong Kong census, the Geo-Community Database, and the Geo-Reference Database, which contain information needed for calculating neighborhood poverty rates and other socioeconomic correlates. Descriptions of the three databases and their accessibility could be found on the websites of Census and Statistics Department (http://www.censtatd.gov.hk/home/index.jsp) and Lands Department (http://www.landsd.gov.hk/en/about/welcome.htm). The geographic unit that we used to indicate a neighborhood was the large street block (LSB). The whole territory of Hong Kong was divided into 1,620 LSBs (mean population = 4,364). The poverty rate for each LSB was calculated as the dependent variable (i.e. population living in households with a combined income under the official poverty line divided by the total population in the LSB, based on household income data from the Hong Kong census).

Following the seminal studies by Wilson [3], Massey and Denton [4], Jargowsky [8], and the more recent studies from Longley [27], Benson, Chamberlin [5], Ali, Partridge [31], and Delang and Lung [29], as well as taking into account Hong Kong’s specific context, we constructed independent variables as follows to proxy the quality of socioeconomic condition (class-based segregation) (1–3), the degree of immigration-based segregation (4), demographic structure (5–8), and neighborhood characteristics influenced by public policies (9–12): 1) unemployment rate; 2) the proportion of people with non-professional jobs; 3) the proportion of people with secondary education or below; 4) the proportion of new Chinese immigrants (i.e. immigrants whose residence was mainland China five years ago); 5) the proportion of single-parent households; 6) the proportion of single-elderly households; 7) dependency ratio (the number of people aged 0–14 and aged 65 and over, divided by the number of people aged 15–64); 8) population density; 9) the proportion of households living in public housing; 101) road density (the length of a neighborhood’s total road network divided by its land area); 11) access to hospitals; and 12) access to physical activities services (indoor games halls/recreation centers/sports centers, sports grounds, public gardens/parks, swimming pools, bowling greens, and tennis courts). The scores for access to hospitals and physical activities were calculated based on the two-step floating catchment area (2SFCA) method [32]; a higher value indicates better service accessibility.

### Spatial and statistical analysis

The exploratory spatial data analysis (ESDA) method was used to investigate the spatial patterning of neighborhood poverty. We used Moran’s *I* to examine spatial dependency, defined as [33]:

$$I=(\frac{n}{\sum_{i}\sum_{j}w_{ij}})(\frac{\sum_{i}\sum_{j}w_{ij}(x_{i}-\bar{x})(x_{j}-\bar{x})}{\sum_{i}{\left(x_{i}-\bar{x}\right)}^{2}})$$

where *x* represents the value of object *i*; *j* represents the index of the units in the research area, of which there are *n* in total; and *w*_*ij*_ is the spatial weight between unit *i* and *j*, which represents the connection between *i* and *j*. Positive Moran’s *I* values suggest that similar values tend to cluster in neighboring areas, whereas negative values mean that high values are frequently accompanied by low values in neighboring areas. In this study, neighborhoods that share a boundary were defined as “neighbors.” The local indicators of spatial association (LISA) map was used to visualize the spatial clusters of the areas of poverty concentration [34]. Four groups can be identified based on the LISA map, including a statistically significant cluster of high values (HH), cluster of low values (LL), a high value surrounded by low values (HL), and a low value surrounded primarily by high values (LH) [34].

To model the relationship between neighborhood poverty rates and its correlates, we conducted two sets of analyses using two different models: ordinary least square (OLS) regression and GWR. The first set of analyses was to identify the global or “city-wide” relationship between neighborhood poverty and potential correlates using the conventional OLS regression model. The regression equation could be written as:

$$y=a_{0}+\sum_{j}x_{ij}a_{j}+\varepsilon_{i}$$ (1)

where *y* is the dependent variable, *x* is the independent variable, *a* is the coefficient of the regression, *i* is the index for the location, *j* is the index for the independent variable, and *ε* is the error term [5, 27]. In the OLS model (1), we assume that the spatial processes accounting for neighborhood poverty levels are the same across the whole study area. That is to say, the relationship between the dependent variable and independent variables are spatially stationary. The global model may hide or mask potential spatial heterogeneity, or spatial non-stationarity, in identifying the correlates of neighborhood poverty rates.

In contrast, the GWR model (2) offers an opportunity to assess the degree to which the association between the dependent variable and independent variables varies across the study area (i.e. a “local-specific” association). This method performs a local model for each area unit (i.e. LSB in the study). This is achieved by constructing a spatial weighting matrix and running a weighted regression for each area unit. The global regression model of OLS above can be reworked toward a local regression model and written as:

$$y_{i}=a_{0i}+\sum_{j}x_{ij}a_{ij}+\varepsilon_{i}$$ (2)

in which the coefficients for each area unit are estimated [35]. The neighboring units of each area unit can be determined using either of the two main kernel types: fixed or adaptive. The fixed type selects an optimal bandwidth for the whole area and all the points that fall within the bandwidth are used in the regression; the adaptive type choses a specified number of nearest neighbors. We used the adaptive approach based on the consideration of the uneven geographic distribution of LSBs in Hong Kong, where the fixed bandwidth approach appears inappropriate. The choice of the optimal number of nearest neighbors was based on that which minimized the Akaike information criterion (AIC) [36]. A spatial statistic tool (“ArcToolBox—Spatial Statistics Tools—Modelling Spatial Relationships—Geographically Weighted Regression”) in ArcGIS version 10.2 was used to perform the analysis. For this study, the optimal number of neighbors was determined to be 465.

This study was approved by the Human Research Ethics Committee for Non-Clinical Faculties, the University of Hong Kong (Ref: EA1508040).

## Results

### Spatial pattern of poverty and independent variables

**Fig 1:**
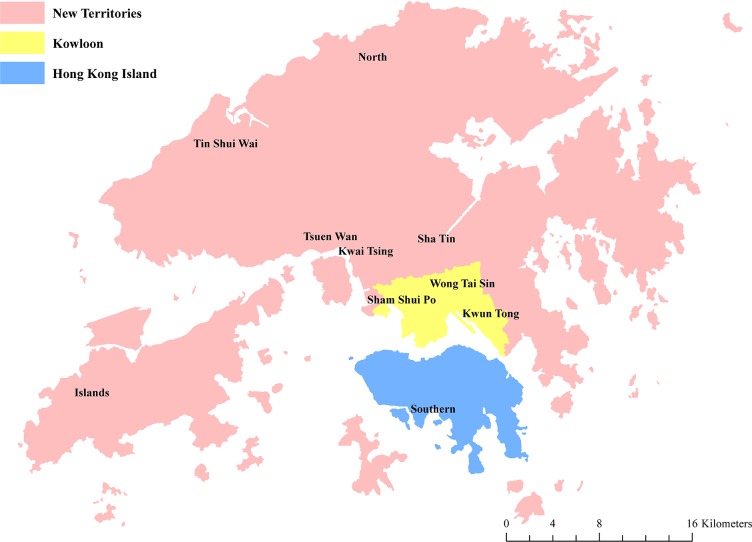
Map of Hong Kong (data in S1 File).

**Fig 2:**
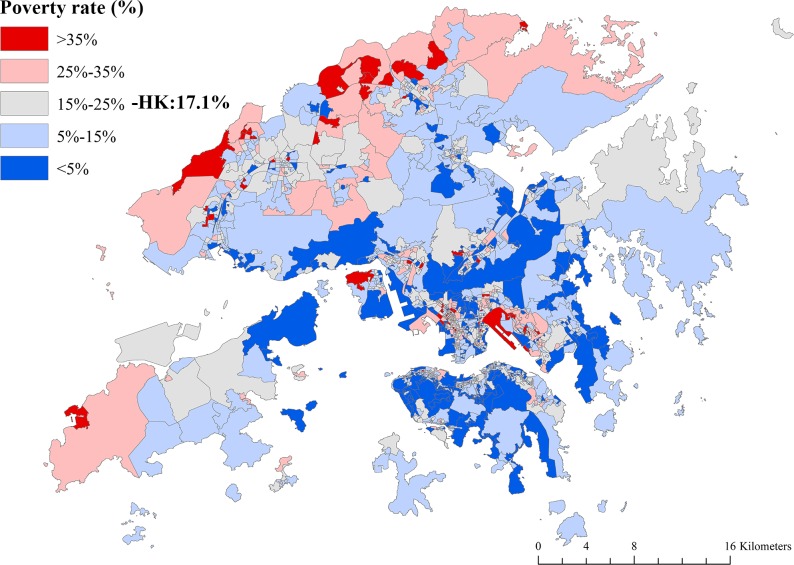
Mapping the poverty rates (%) of 1,620 LSBs in Hong Kong, 2011 (data in S2 File).

**Fig 3:**
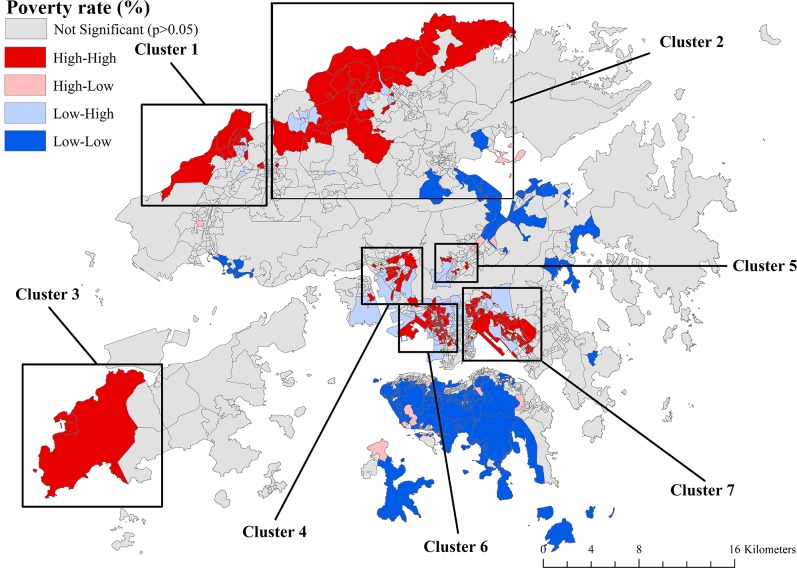
LISA map of the poverty rates of 1,620 LSBs, Hong Kong, 2011 (data in S3 File).

Fig 1 is the map of Hong Kong, with three main regions, i.e. Hong Kong Island, Kowloon, and the New Territories. Fig 2 shows the geographic patterning of poverty rates in Hong Kong, identifying many poorer neighborhoods in the New Territories and Kowloon and only a few poorer neighborhoods in Hong Kong Island. Moran’s *I* was 0.11 (*p* < 0.001), indicating a low degree of overall spatial clustering (i.e. non-randomness) in neighborhood poverty rates across Hong Kong. The LISA map of neighborhood poverty rates shows four different types of cluster (Fig 3). Areas highlighted in red had a relatively high poverty rate with neighboring areas also showing relatively high concentration of poverty (i.e. “hot spots”). The LISA map allows the identification of seven apparent regions with concentrated, high poverty rates in Hong Kong: five (clusters 1, 2, 3, 4, and 7) in the New Territories, and two (clusters 5 and 6) in Kowloon. By contrast, the majority of Hong Kong Island showed a “cold spot” with clustering of low-poverty areas.

**Fig 4:**
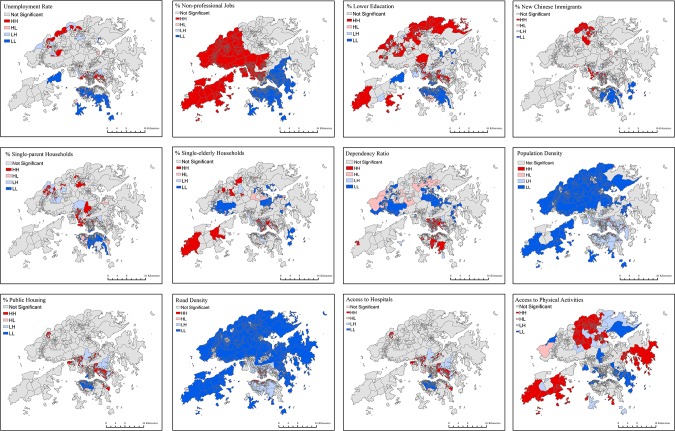
LISA maps of the independent variables of 1,620 LSBs in Hong Kong, 2011 (data in S4 File).

LISA maps also indicate spatial heterogeneity of the independent variables (Fig 4). For instance, hot spots for persons with lower education were mainly found in the New Territories and Kowloon, whereas most areas of Hong Kong Island were cold spots. High population density and road density were concentrated in Kowloon and the north of Hong Kong Island, while low values were clustered in most areas of the New Territories.

### City-wide and local-specific correlates of neighborhood poverty

Table 1 summarizes the results of the spatial regression analysis based on the OLS (1) and GWR (2) models. The OLS model showed that nine variables were associated with neighborhood poverty rates in Hong Kong at the city-wide level. Specifically, with the exception of population density, all other variables of socioeconomic condition, immigration-based segregation, and demographic structure showed a positive association with the neighborhood poverty rate. In terms of public policy-related variables, the proportion of households living in public housing was positively associated with poverty rates; by contrast, there was no statistical evidence for an association between neighborhood poverty rate with access to hospitals or physical activities. Road density was positively associated with poverty rate, contrary to our expectation of a negative association. The variance inflation factor (VIF) values for all variables were lower than 10, indicating no strong evidence for multicollinearity [37]. The adjusted *R*^2^ of the OLS regression model was 0.656.

**Table 1:** Comparison of OLS and GWR for neighborhood poverty rate, Hong Kong, 2011.

	OLS		GWR					LSB%	
	Adjusted *R*^2^ = 0.656		Adjusted *R*^2^ = 0.726						
	AIC = 2892.4		AIC = 2769.0						
	Coefficient	VIF	Min	Lower Quartile	Median	Upper Quartile	Max	Negative association with poverty	Positive association with poverty
**Variables**									
**Unemployment rate**	0.168***	1.215	0.003	0.117	0.155	0.183	0.231	0.00	93.82
**% Non-professional jobs**	0.180***	4.992	-0.066	0.071	0.188	0.300	0.449	0.00	55.81
**% Lower education**	0.321***	5.497	-0.019	0.231	0.377	0.463	9.566	0.00	89.39
**% New Chinese immigrants**	0.137***	1.187	0.004	0.053	0.083	0.159	0.303	0.00	53.87
**% Single-parent households**	0.080***	1.251	-0.067	0.027	0.066	0.124	0.179	0.00	45.32
**% Single-elderly households**	0.080***	1.471	-0.104	0.005	0.081	0.115	0.204	0.00	45.32
**Dependency ratio**	0.090***	1.339	-0.072	0.025	0.077	0.133	0.228	0.00	48.75
**Population density**	0.015	1.496	-0.061	-0.010	0.020	0.044	0.126	0.00	2.06
**% Public housing**	0.153***	1.573	-0.036	0.082	0.145	0.210	0.303	0.00	68.29
**Road density**	0.055**	1.644	-0.158	-0.143	0.023	0.082	0.178	5.12	17.04
**Access to hospitals**	-0.010	1.135	-0.257	-0.093	0.076	0.206	0.465	11.30	18.23
**Access to physical activities**	0.011	1.103	-0.391	-0.095	-0.007	0.085	0.608	0.00	0.81

**: *p* < 0.01

***: *p* < 0.001.

**Fig 5:**
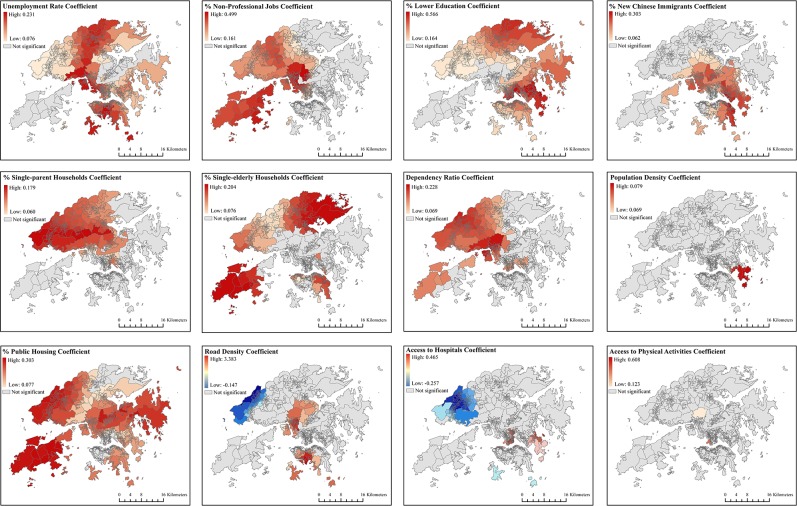
Local regression coefficient values from the GWR model in Hong Kong, 2011 (data in S4 File).

The results of the GWR model are shown in Fig 5 and Table 1. Fig 5 shows spatial variations in regression coefficients for each of the independent variables. Table 1 shows the distribution of regression coefficients and the percentage of LSBs presenting statistically significant negative or positive associations with poverty rate for each of the independent variables. The results of the GWR model show the spatial heterogenity of the association with poverty rate for some variables. For example, spatial variation in the association with poverty rate was particularly apparent for access to hospitals, with 11.30% of LSBs having a negative association (mainly in the northwest of the New Territories or around the Tin Shui Wai area; Fig 5 and Fig 1) and 18.23% of LSBs showing a positive association (mainly in Kowloon; Fig 5). Road density also showed negative associations with poverty rates in 5.12% of LSBs (mainly in the northwest of the New Territories or around the Tin Shui Wai area; Fig 5 and Fig 1) and positive associations in 17.04% of LSBs (mainly in the south of Hong Kong Island and some suburban areas close to Kowloon; Fig 5).

**Fig 6:**
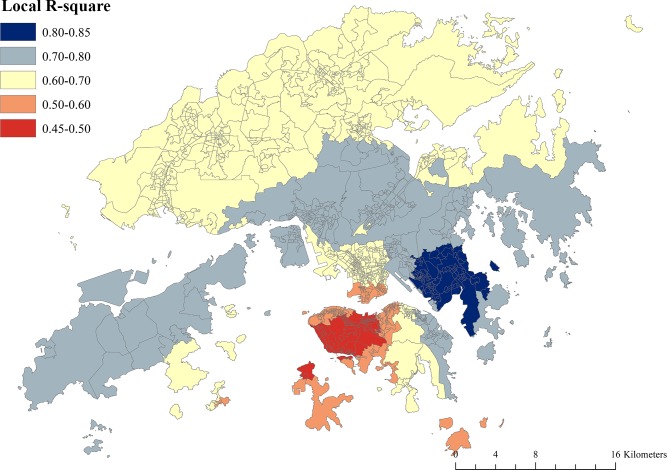
Local *R*^2^ calculated from the GWR model of the correlates of neighborhood poverty rate in Hong Kong (data in S5 File).

The results of the OLS and GWR models were more consistent for unemployment rate and the proportion of population with lower education than other variables–significantly positive associations with poverty rate were found in more than 85% of LSBs. For other variables that showed a positive association with poverty rate in the OLS model, the percentage of LSBs showing a positive association in the GWR model ranged between 45% (the proportion of single-parent households and single-elderly households) and 68% (the proportion of households living in public housing) (Table 1), whilst the spatial patterning of positive associations varied by variable (Fig 5). For example, the proportion of single-parent households showed a positive association with poverty in most of the western areas of the New Territories and parts of Kowloon, whilst a positive association of the proportion of single-elderly households with poverty was found in a number of areas in Hong Kong Island, some areas in eastern Kowloon, and most north and northwestern parts of the New Territories. The adjusted *R*^2^ of the GWR regression model was 0.726, which indicated a 7% improvement over the OLS model (adjusted *R*^2^ = 0.656). Fig 6 shows the variation of adjusted *R*^2^ across Hong Kong.

### Characteristics of poverty clusters

Table 2 shows the proportions of LSBs in each of the seven clusters where potential correlates were significantly associated with neighborhood poverty rates. In each of the seven poverty clusters, two variables–the proportion of households living in public housing and dependency ratio–were significantly associated with neighborhood poverty rates in more than half the LSBs. The proportion of non-professional workers was also associated with neighborhood poverty rates in each of the poverty clusters (significant in more than 80% of LSBs) with only one exception: cluster 7 (significant in only 17% of LSBs) in Kowloon. Unemployment rate and the proportion of single-parent households were also significantly associated with neighborhood poverty rates in five of the seven clusters. Access to physical activities was not related to neighborhood poverty rates in any of the seven clusters and population density was significant in only 5% of LSBs in poverty cluster 7 (Table 2).

**Table 2:** Proportion of LSBs where potential correlates were significantly associated with neighborhood poverty rates in seven poverty clusters, Hong Kong, 2011.

	New Territories					Kowloon	
Variables (%)	Cluster 1	Cluster 2	Cluster 3	Cluster 4	Cluster 5	Cluster 6	Cluster 7
**Unemployment rate**	100 (+)	100 (+)	non-significant	100 (+)	67 (+)	100 (+)	99 (+)
**Non-professional jobs**	100 (+)	80 (+)	100 (+)	100 (+)	100 (+)	100 (+)	17 (+)
**Lower education**	100 (+)	100 (+)	non-significant	non-significant	non-significant	100 (+)	100 (+)
**New Chinese immigrants**	non-significant	non-significant	non-significant	100 (+)	100 (+)	98 (+)	90 (+)
**Single-parent households**	100 (+)	100 (+)	non-significant	100 (+)	83 (+)	non-significant	58 (+)
**Single-elderly households**	100 (+)	100 (+)	100 (+)	non-significant	non-significant	non-significant	9 (+)
**Dependency ratio**	100 (+)	84 (+)	100 (+)	100 (+)	50 (+)	76 (+)	53 (+)
**Population density**	non-significant	non-significant	non-significant	non-significant	non-significant	non-significant	5 (+)
**Public housing**	100 (+)	80 (+)	100 (+)	100 (+)	100 (+)	87 (+)	90 (+)
**Road density**	100 (-)	non-significant	non-significant	100 (+)	33 (+)	non-significant	non-significant
Access to hospitals	100 (-)	8 (+)	non-significant	non-significant	non-significant	1 (+)	29 (+)
Access to physical activities	non-significant	non-significant	non-significant	non-significant	non-significant	non-significant	non-significant

Note: in each LSB of the seven clusters, the association between neighborhood poverty rates and potential correlates can be: “positively significant,” “non-significant,” or “negatively significant.” In the table, the percentages are the proportions of LSBs that were significantly associated with neighborhood poverty rate.

“+” indicates positively significant association and “-” indicates negatively significant association.

“Non-significant” indicates that there was no significant association between neighborhood poverty rates and the potential correlates in any LSB in a particular cluster.

Some distinct characteristics of poverty concentration were identified in the seven poverty clusters (Figs 3 and 5). The proportion of people with lower education showed a positive association with neighborhood poverty rates in the north of the New Territories (cluster 1 and cluster 2) and two inner-city areas, Sham Shui Po (cluster 6) and Wong Tai Sin-Kwun Tong (cluster 7) in Kowloon, while no such association was found in two New Towns close to Kowloon, Kwai Tsing-Tsuen Wan (cluster 4) and Sha Tin (cluster 5). The proportion of new Chinese immigrants was an important correlate of poverty (significant in more than 90% of LSBs) in Kowloon (cluster 6 and cluster 7) and in southern areas of the New Territories (cluster 4 and cluster 5) close to Kowloon, but it was not associated with neighborhood poverty rates in any LSBs of the poverty clusters in northern or western areas of the New Territories (cluster 1, cluster 2, and cluster 3). The proportion of single-elderly households was a correlate of poverty in all LSBs within the poverty clusters in suburban areas (cluster 1, cluster 2, and cluster 3) but not associated with poverty in the poverty clusters in Kowloon and the south of the New Territories close to Kowloon. Road density and access to hospitals were significant correlates of poverty only in cluster 1 in the northwest of the New Territories far from inner-city areas (Table 2).

## Discussion

The spatial patterning of poverty and its correlates in Hong Kong showed certain characteristics distinct from those found in Western cities. Several clusters with concentrated poverty were identified in both inner-city and suburban areas of Hong Kong. Neighborhood socioeconomic conditions contributed more than immigration-based segregation, demographic structure, and public policy to explaining the spatial patterning of poverty at the city-wide level based on results from the OLS model. However, the GWR model showed that the association of poverty with some correlates varied across areas and different poverty clusters, suggesting that poverty concentration takes different forms at the local-specific level. In order to mitigate poverty in Hong Kong, more focused poverty alleviating measures are needed to provide effective responses to the poverty situation at the local level.

### Strengths and limitations

To the best of our knowledge, this is the first detailed spatial analysis of poverty at the small-area level in Hong Kong. In contrast to previous spatial studies conducted at a larger geographic scale, such as TPU [29, 38], we used detailed small-area data at the LSB level to reduce the effect of heterogeneity within the area unit. A broad array of variables were investigated for their association with neighborhood poverty. The comprehensive structure of the models made the analysis more systematic than studies which only focused on specific dimensions such as socioeconomic conditions. Moran’s *I* was used to examine spatial dependency of neighborhood poverty and LISA map was used to visualize the spatial clusters of the areas with high poverty level. The analysis was conducted on both the city-wide and local-specific levels in order to avoid the heterogeneity of associations being masked as is found in studies only conducted at the general level [5].

While the purpose of this research was to uncover spatial variations in rate of poverty (income-based deprivation) and its correlates, it did not investigate the poverty experienced by local residents. Second, this study focused on different forms of poverty concentration at single time point, and summarized the characteristics of each poverty cluster in a general, descriptive way. Further studies investigating spatial-temporal transformation of multiple deprivation can provide a more comprehensive picture of urban vulnerability in Hong Kong. Third, certain measures can be improved by using more detailed data and complex models. For example, we used road density to proxy transport accessibility, while transport accessibility has many dimensions apart from access to road networks, such as access to public transportation e.g. bus or subway stations, or the cost of travel in daily activity.

### Spatial patterning of poverty

Our results show that the spatial distribution of poverty in Hong Kong is not homogeneous and that areas with a high degree of poverty are clustered systematically. This finding is generally similar to findings pertaining to large, developed Western cities [39–41]. However, while it has been seen in the West that poverty is mainly concentrated in inner-city areas [3], the concentration of high poverty levels are found in both inner-city and suburban Hong Kong. The pattern is related to the policies of New Town development over the past several decades and the public housing policy pursued by the Hong Kong government, which built public housing in many new towns in the New Territories [29, 42]. It was perhaps not surprising that poverty clusters were only identified in Kowloon and the New Territories but not on Hong Kong Island, where there was low level of public housing as a result of the government’s public housing plans [43].

### Correlates of poverty and different forms of poverty concentration

The findings from the comparison between the city-wide and local-specific models provides strong evidence that the correlates of neighborhood poverty rates vary spatially in their effects across Hong Kong.

On the city-wide level, our results support the arguments put forward by both Wilson [3] and Massey and Denton [4] that class-based and race-based segregation (immigration-based segregation in Hong Kong) are both important to poverty concentration. Some studies note that people living in economically or racially isolated areas have weaker social networks than those living in more economically and racially integrated areas, which would further deteriorate the poverty concentration as social networks a person has may play an important role in their ability to gain opportunities [9, 44]. Our findings also support the argument by Jargowsky [8] that findings of class-based segregation and race-based segregation are not mutually exclusive. However, unlike previous Jargowsky [10] studies devoted to identifying which factors are more important in explaining poverty concentration, our study followed Cooke [2] to investigate whether different factors play different roles in poverty concentration in different areas. The results of local-specific analysis showed that socioeconomic condition was a more common correlate of neighborhood poverty across different areas in Hong Kong, while immigration-based segregation mainly contributed to poverty concentration in inner-city areas and certain suburban areas in the New Territories close to Kowloon. Similar to findings from the United States, this was probably due to the fact that new arrivals tend to reside in districts close to central region where the cost of living is relatively low [19] and there are more job opportunities.

In terms of other factors, including demographic structure and public policy, most of our findings at the city-wide level are similar to those of previous studies, while our research makes a contribution to knowledge at the local-specific level. Specifically, in keeping with previous studies [5], our results suggest that demographic structure, especially vulnerable groups, plays a significant role in explaining the spatial variation of poverty. The finding that the proportion of single-parent households is a significant correlate in the clusters in Kowloon and the New Territories mirrors the results of Jargowsky [8], who found that one of the major features of poverty concentration areas is a higher proportion of female-headed households. Although Delang and Lung [29] suggested that public housing does not necessarily cause concentrated poverty, his conclusion was drawn at the larger geographic scale of the TPU. Our results suggest that at the finer level of the LSB, public housing is an important correlate of neighborhood poverty. Regarding public services, our local-specific analyses showed that they were very important correlates in certain areas of Hong Kong. One of these areas showing a striking pattern is the region around Tin Shui Wai (poverty cluster 1), which is relatively isolated from the city center with limited local job opportunities [45] and showed strong negative associations of local poverty rate with road density and access to hospitals, suggesting a marked local unmet need for access to transportation and health services.

### Implications

Our results show that there are marked geographic variations in neighborhood poverty rates in Hong Kong, and the correlates of high-poverty neighborhoods vary across poverty clusters by location. This suggests that heterogeneity masked in city-wide analysis may lead to inappropriate poverty alleviation measures and prevent the optimization of resource allocation. Policymaking should take into account correlates of poverty at both the city-wide and local-specific levels. This depends upon whether the association between neighborhood poverty rates and their correlates is shown to be spatially stationary. If the correlates of poverty are spatially stationary across a city, it is appropriate to make city-wide policies. Based on the findings from this study, for example, Hong Kong’s city-wide strategies could include creating educational and employment opportunities. By contrast, for spatially non-stationary correlates of poverty, “one-size-fits-all” poverty alleviation policies should be replaced by local-specific and targeted approaches that take into account local contexts. Based on findings from this study, for example, there could be a stronger need for access to transportation and health services in Tin Shui Wai (poverty cluster 1) than in other areas. Similarly, efforts to improve new Chinese immigrants’ social participation could be more helpful in alleviating poverty in inner-city clusters than in other clusters in New Territories.

## Supporting information

S1 File Data for Fig 1. (XLSX)

S2 File Data for Fig 2. (XLSX)

S3 File Data for Fig 3. (XLSX)

S4 File Data for Fig 4 and Fig 5. (XLSX)

S5 File Data for Fig 6. (XLSX)
